# Evaluation of humoral and cellular response to four vaccines against COVID-19 in different age groups: A longitudinal study

**DOI:** 10.3389/fimmu.2022.1021396

**Published:** 2022-10-31

**Authors:** Giorgio Fedele, Filippo Trentini, Ilaria Schiavoni, Sergio Abrignani, Guido Antonelli, Vincenzo Baldo, Tatjana Baldovin, Alessandra Bandera, Filippa Bonura, Pierangelo Clerici, Massimo De Paschale, Francesca Fortunato, Andrea Gori, Renata Grifantini, Giancarlo Icardi, Tiziana Lazzarotto, Vittorio Lodi, Claudio Maria Mastroianni, Andrea Orsi, Rosa Prato, Vincenzo Restivo, Rita Carsetti, Eva Piano Mortari, Pasqualina Leone, Eleonora Olivetta, Stefano Fiore, Angela Di Martino, Silvio Brusaferro, Stefano Merler, Anna Teresa Palamara, Paola Stefanelli

**Affiliations:** ^1^ Department of Infectious Diseases, Istituto Superiore di Sanità, Rome, Italy; ^2^ Center for Health Emergencies, Bruno Kessler Foundation, Trento, Italy; ^3^ Dondena Centre for Research on Social Dynamics and Public Policy, Bocconi University, Milan, Italy; ^4^ Istituto Nazionale Genetica Molecolare, Padiglione Romeo ed Enrica Invernizzi, Milan, Italy; ^5^ Department of Clinical Sciences & Community Health, University of Milan, Milan, Italy; ^6^ Department of Molecular Medicine, AOU Policlinico Umberto I, Sapienza University, Rome, Italy; ^7^ Laboratory of Hygiene and Applied Microbiology, Hygiene and Public Health Unit, Department of Cardiac Thoracic and Vascular Sciences and Public Health, University of Padova, Padova, Italy; ^8^ Infectious Diseases Unit, Foundation IRCCS Ca’ Granda Ospedale Maggiore Policlinico, Milan, Italy; ^9^ Centre for Multidisciplinary Research in Health Science (MACH), University of Milano, Milan, Italy; ^10^ Department of Health Promotion, Mother and Child Care, Internal Medicine and Medical Specialties, University of Palermo, Palermo, Italy; ^11^ Microbiology Unit, Azienda Socio Sanitaria Territoriale (ASST) Ovest Milanese, Milan, Italy; ^12^ Hygiene Unit, Policlinico Riuniti Foggia Hospital, Department of Medical and Surgical Sciences, University of Foggia, Foggia, Italy; ^13^ Hygiene Unit, IRCCS Ospedale Policlinico San Martino Genova, and Department of Health Sciences, University of Genoa, Genoa, Italy; ^14^ Microbiology Unit, IRCCS Azienda Ospedaliero-Universitaria di Bologna, Bologna, Italy; ^15^ Section of Microbiology, Department of Experimental, Diagnostic and Specialty Medicine, University of Bologna, Bologna, Italy; ^16^ Occupational Health Unit, IRCCS Azienda Ospedaliero-Universitaria di Bologna, Bologna, Italy; ^17^ Department of Public Health and Infectious Disease, AOU Policlinico Umberto I, Sapienza University, Rome, Italy; ^18^ B Cell Lab, Immunology Research Area, Bambino Gesù Children’s Hospital, IRCCS, Rome, Italy; ^19^ National Center for Global Health, Istituto Superiore di Sanità, Rome, Italy; ^20^ Istituto Superiore di Sanità, Rome, Italy

**Keywords:** COVID-19, vaccines, serology, cell-mediated immunity, B-cell memory

## Abstract

To date there has been limited head-to-head evaluation of immune responses to different types of COVID-19 vaccines. A real-world population-based longitudinal study was designed with the aim to define the magnitude and duration of immunity induced by each of four different COVID-19 vaccines available in Italy at the time of this study. Overall, 2497 individuals were enrolled at time of their first vaccination (T0). Vaccine-specific antibody responses induced over time by Comirnaty, Spikevax, Vaxzevria, Janssen Ad26.COV2.S and heterologous vaccination were compared up to six months after immunization. On a subset of Comirnaty vaccinees, serology data were correlated with the ability to neutralize a reference SARS-CoV-2 B strain, as well as Delta AY.4 and Omicron BA.1. The frequency of SARS-CoV-2-specific CD4+ T cells, CD8+ T cells, and memory B cells induced by the four different vaccines was assessed six months after the immunization. We found that mRNA vaccines are stronger inducer of anti-Spike IgG and B-memory cell responses. Humoral immune responses are lower in frail elderly subjects. Neutralization of the Delta AY.4 and Omicron BA.1 variants is severely impaired, especially in older individuals. Most vaccinees display a vaccine-specific T-cell memory six months after the vaccination. By describing the immunological response during the first phase of COVID-19 vaccination campaign in different cohorts and considering several aspects of the immunological response, this study allowed to collect key information that could facilitate the implementation of effective prevention and control measures against SARS-CoV-2.

## Introduction

The response to the SARS-CoV-2 pandemic relied on the development, testing, and deployments of COVID-19 vaccines. In Italy, the vaccination campaign started from late December 2020. Two different mRNA vaccines, Pfizer BioNTech BNT162b1 (Comirnaty) and Moderna mRNA-1273 (Spikevax), and two adenoviral vector-based vaccines, AstraZeneca ChAdOx1-S (Vaxzevria) and Janssen Ad26.COV2.S have been licensed for their use (reviewed in (1)). Reports on vaccine efficacy in preventing disease are available (2–5).

Old and frail individuals have been identified with the highest risk of negative health outcomes after SARS-CoV-2 infection. An association between poor prognostic outcomes and advancing age has been established (6–9). A meta-analysis of studies on the effect of age difference on vaccine safety and efficacy concluded that immunogenicity of COVID-19 vaccines is lower in older adults (10). Remarkably, it has been shown that after the administration of COVID-19 vaccine older individuals have lower antibody response than younger subjects (11) and that vaccine-induced immune response is strongly increased by a third booster dose (12, 13).

Available data suggest that coordinated functions of different branches of the innate and adaptive immunity provide multiple mechanisms of protection against COVID-19. Although the levels of neutralizing antibodies have been suggested to correlate with protection against infection (14, 15) both T and B cell memory response contribute to protective immunity (16, 17).

Monitoring of the COVID-19 immunization campaigns represents a unique opportunity to collect and analyse immune responses in a longitudinal real-world study. The present study was designed with the main aim of establishing the magnitude and duration of immunity induced by each of the four different vaccines available in Italy at the time of the study. To this end, healthy adults aged less than 65 years of age and frail elderly aged over 65 years of age were enrolled. Vaccine-specific antibody responses induced over time were compared up to six months after the first vaccine dose. On a subset of Comirnaty vaccinees, serology data were correlated with the ability to neutralize an ancestral SARS-CoV-2 strain, as well as Delta and Omicron sub-lineages. To complete the evaluation of the response of the adaptive immune system to vaccination, the frequency of SARS-CoV-2-specific CD4+ T cells, CD8+ T cells, and memory B cells induced by the four different vaccines was assessed six months after the first vaccine dose. Hereby, we report the results obtained during the first part of the monitoring study concerning immune responses up to six months after the primary two-dose vaccination schedule.

## Methods

### Study design

A multicentre longitudinal cohort study was designed to monitor immune responses in individuals vaccinated with the COVID-19 vaccines that have been in use in Italy: Comirnaty (Pfizer); Spikevax (Moderna); Vaxzevria (Astra Zeneca); Ad26.COV2-S (Janssen).

Two cohorts, adults ≤ 65 years of age and frail subjects > 65 years of age with at least two co-morbidities associated with increased risk of severe COVID-19 (listed in **Supplementary Table 1**), were enrolled in eight collaborating centres from seven Italian regions at time of their first COVID-19 vaccine dose. The list of participating centres is shown in **Supplementary Table 2**.

Venous blood withdrawal was planned at first vaccination (T0), one month after the completion of the primary vaccine series (T1), and six months after the first vaccine dose (T2). The study is still ongoing, and a third venous blood withdrawal is planned by twelve months after the first vaccine dose (T3).

At the enrolment, a questionnaire was administered to subjects who agreed to enter the study to collect demographic and clinical data, including a previous COVID-19 diagnosis, together with the informed consensus form.

To measure possible exposure to natural SARS-CoV-2 infection, IgG levels against the Nucleocapsid (N) protein were measured at each time-point.

### Serum preparation and storage

Blood samples (5 ml) were collected in Serum Separator Tubes (BD Diagnostic Systems, Franklin Lakes, NJ, USA) and centrifuged at room temperature at 1600 rpm for 10 min. Two serum aliquots were transferred to 2ml polypropylene, screw cap cryo tubes (Nunc™, Thermofisher Scientific, Waltham, MA USA), immediately frozen at -20°C and thereafter stored at -80°C. Frozen sera were shipped to the Department of Infectious Diseases at Istituto Superiore di Sanità (ISS), in dry ice following biosafety shipment condition. Upon arrival serum samples were immediately stored at -80°C.

### SARS-CoV-2 IgG immunoassays

Sera were evaluated centrally at ISS by the DiaSorin Liaison SARS-CoV-2 trimericS IgG assay on the LIAISON^®^ XL chemiluminescence analyzer (DiaSorin, Saluggia, VC, Italy). The assay range is up to 2080 Binding Antibody Units (BAU/mL). According to manufacturer’s instructions, values ≥ 33.8 BAU/mL were interpreted as positive. If the results were above the assay range, samples were automatically diluted 1/20 and testing was repeated.

Anti-Nucleocapsid (N) IgG were measured at T0, T1, T2 after serum preparation by the collaborating centres. The anti-N IgG Elecsys (Roche Diagnostics, Monza, Italy); Anti-N IgG iFlash (Pantec, Torino, Italy) and anti-N IgG Architect (Abbott Diagnostics, Chicago, IL, USA) were used, data were interpreted according to manufacturers’ instructions.

### SARS-CoV-2 neutralizing antibody assay

SARS-CoV-2 isolates belonging to B (considered as reference strain in this study), AY.4 (Delta) and BA.1 (Omicron) lineages were incubated with two-fold serial dilutions of serum samples starting at 1:8 dilution in E MEM culture medium (Sigma Aldrich, Merck Life Science, Milan, Italy) supplemented with 1X penicillin/streptomycin (Corning, Glendale, AZ, USA) and 2% foetal bovine serum (Corning) in 96-well plates. Virus (100 TCID50) and serum mixture was incubated at 37°C for 1 hour. After this incubation 22,000 cells per well were added and incubated at 37°C for 5 days. The neutralization titer was calculated and expressed as microneutralization titer 50 (MNT50), i.e., the serum dilution capable of reducing the cytopathic effect to 50%.

### Assessment of SARS CoV-2 Spike-specific T-cell response

Spike protein-specific T-cell responses were measured by stimulating patients’ peripheral blood mononuclear cells (PBMCs) with a pool of overlapping peptides covering the immunodominant domains of the ancestral Spike protein (Miltenyi, Bergisch Gladbach, Germany). After overnight stimulation, cells were incubated with Live/Dead fixable violet dead cell stain kit used to exclude dead cells from the analyses (Thermo Fisher Scientific). Cells were then fixed and permeabilized using Cytofix/Cytoperm Fixation/Permeabilization Solution Kit (ThermoFisher) and stained with a predetermined optimal concentration of fluorochrome-conjugated Abs: anti-CD3-APC-H7, anti-IL-2-FITC, anti-TNFα PE-Cy7 (all from BD Biosciences, Franklin Lakes, NJ, USA), anti-IFN-γ-PerCP-Cy5.5 (both from Biolegend, San Diego, CA, USA), anti-CD8-APC (eBiosciences, Thermo Fisher Scientific). Cells were then acquired by a Gallios Flow Cytometer (Beckman Coulter, Indianapolis, IN, USA) and data analyzed with Kaluza Analysis software (Beckman Coulter). Unstimulated PBMCs were used as negative control. As a positive control, the non-specific superantigen SEB was added at 100 ng/ml (Sigma-Aldrich). Frequencies of cytokine producing cells were calculated after subtraction of cytokine positive cells in the relative negative control tube, i.e., unstimulated sample. Gating strategy is shown in **Supplementary Figure 1**. A Boolean gating strategy was used to identify polyfunctional T cells.

### Assessment of SARS CoV-2 Spike-specific B-cell memory response

Detection of antigen-specific memory B cells (MBC) was performed as previously published (17, 18). Briefly, two aliquots of biotinylated recombinant ancestral SARS-CoV-2 Spike (S1+S2, R&D Systems, Minneapolis, MN, USA) were individually multimerized with fluorescently labelled streptavidin-PE, and streptavidin-BUV395. The B.1.617.2 (Delta) Spike (R&D Systems) was labelled with streptavidin-FITC at 4°C for one hour. Streptavidin-PE-Cy7 (BD Biosciences) was used as a decoy probe to gate out streptavidin-binding non-specific B-cells. Around 4x10^5^ previously frozen PBMC samples were prepared and stained with antigen probe cocktail containing 100ng Spike per probe (total 300ng) and 2ng streptavidin-PE-Cy7 at 4°C for 30 min to ensure maximal staining quality. Surface staining was performed with labelled-antibodies in brilliant buffer at 4°C for 30 min. Spike-specific memory B cells were identified in the CD19+CD24+CD27+ memory B cell (MBC) population as Spike++ (binding SARS-CoV-2 Spike labelled with PE and BUV-395). Among Spike++ MBCs, those specific for AY.4 (Delta) were also detected (**Supplementary Figure 2**). Samples were acquired on FACS LSRFortessa (BD) and analysed using FlowJo10.7.1 (BD). Limit of detection (LOD) and limit of quantification (LOQ) were calculated as previously reported (19, 20).

### Statistical analysis

Using a log-linear regression model, we investigated the association between the geometric mean anti- SARS-CoV-2 trimeric S IgG titers at 1 month and some covariates of interest: type of administered vaccine, age group, sex, and a categorical variable indicating whether the response at first vaccination was below or above the positivity threshold of 33.8 BAU/mL. Due to the skewness of the distribution of the anti- SARS-CoV-2 trimeric S IgG titers, we considered the log-transformation of the dependent variable in the model.

We further investigated the variation in the decline of the geometric mean anti- SARS-CoV-2 trimeric S IgG titers between 1 and 6 months after vaccination through two mixed effect linear models conducted separately for the two age groups. The dependent variable was the log-transformed anti- SARS-CoV-2 trimeric S IgG titer at 1 and 6 months, and the covariates were time, type of vaccine, and the categorical variable on the response at time T0. A random effect was considered for the subjects enrolled in the study, and an interaction between time and type of vaccine was introduced to account for heterogeneity in the decline of the geometric mean anti- SARS-CoV-2 trimeric S IgG among different types of vaccine administered.

## Results

### Study sample

The enrolment of study participants began in February 2021 and ended in September 2021. Overall, 2497 individuals were enrolled at time of their first vaccination (T0); nine subgroups were defined, based on vaccine type and age/comorbidities (**Supplementary Figure 3**). The final size of the different subgroups was affected by enrolment procedures. Only a few centres were able to start the enrolment early in February and March 2021, when the national vaccination campaign was focused on older age groups. In March 2021, a circular by the Italian Ministry of Health (Nr. 0026246-11/06/2021) changed reccomendations for the use of Vaxzevria. The first two vaccines available were Comirnaty and Vaxzevria; Spikevax, and the Janssen Ad26.COV2 vaccine had a later approval.

### Anti- SARS-CoV-2 trimeric S IgG titer after vaccination

During the study, several individuals dropped out of the study. Final analysis was performed on 1519 of the 1530 subjects whose blood samples were available at all three time points (**Supplementary Figure 3**), since 11 were excluded having received a single vaccine dose instead of the two-dose regimen required. A total of 118 participants (7.8%) had a COVID-19 diagnosis before vaccination, of those, 65 received a single vaccine dose, whereas 53 had two doses.

**Figure 1:**
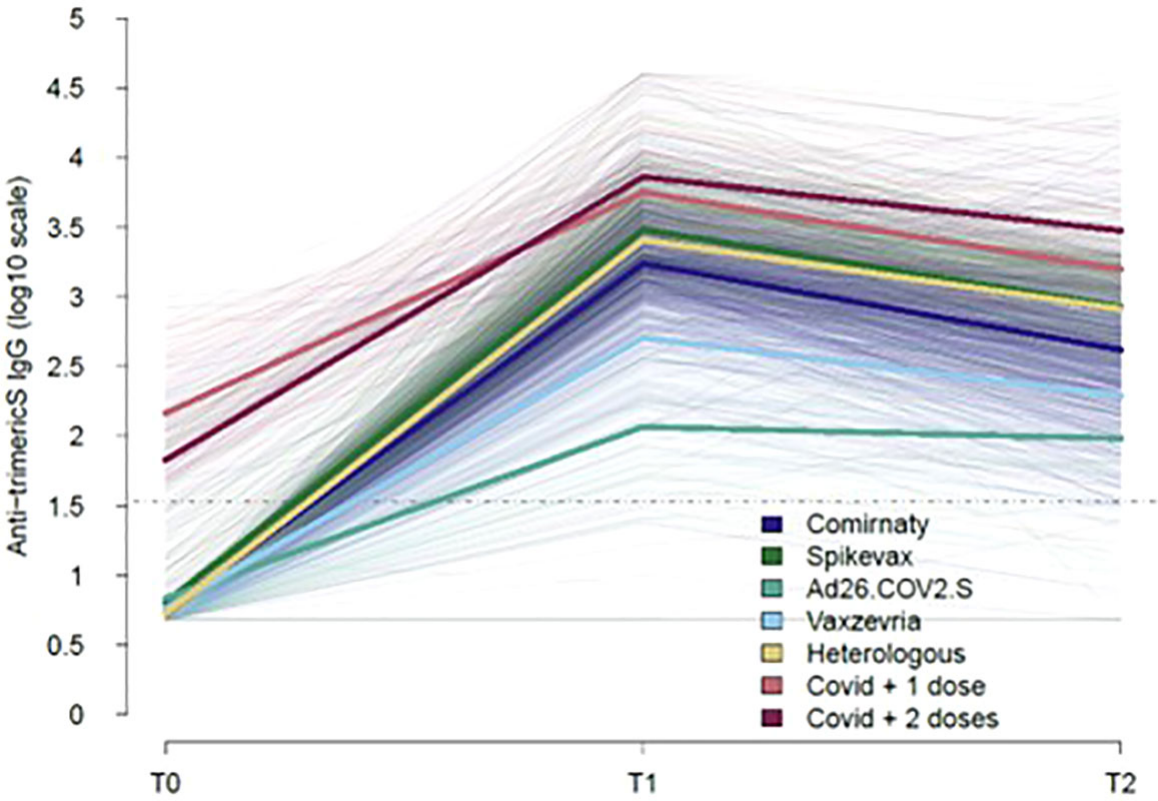
Kinetics of COVID-19 vaccine-induced IgG over time. The trajectories of serum anti-trimeric-Spike IgG (BAU/ml) for all the subjects analysed, stratified by type of vaccine or previous COVID-19 diagnosis are shown (thin lines). Mean values in each group are indicated (thick lines). The dotted line represents the positivity cut-off of the serological assay (33.8 BAU/ml).

**Figure 1** shows the longitudinal trajectories of anti-trimericS IgG in the total sampled population stratified by the type of vaccine received or previous COVID-19 diagnosis (ex-COVID). Overall, anti-trimericS IgG peaked one month after primary vaccination (T1), thereafter declining with significantly lower levels 5 months later (T2) (Wilcoxon Signed-Rank Test p-value <0.001), with the exception of Ad26.COV2 vaccine recipients for whom IgG levels remained stable. Among COVID-19 naïve subjects, mRNA vaccines induced a better response, the highest geometric mean titers (GMT) were reached with the Spikevax vaccine. The Ad26.COV2.S vaccine induced the lowest antibody response. The HV regimen (first dose Vaxzevria and second dose Comirnaty or Spikevax) induced a significantly higher response compared with two doses of Vaxzevria at both T1 and T2 (Wilcoxon Signed-Rank Test p-value <0.001). Higher antibody titers were measured in ex-COVID individuals, either receiving one single vaccine dose or two doses, at all the timepoints analysed (Wilcoxon Signed-Rank Test p-value <0.001).

To avoid the confounding effect of a pre-existing immunity, serological data stratified by vaccine type and age-group were analysed separately among COVID-19 naïve vaccinees (**Table 1**) and ex-COVID subjects (**Table 2**). In COVID-19 naïve subjects the hierarchy in anti-S IgG levels at T1 and T2 was Spikevax > Comirnaty >Vaxzevria > Ad.26.CoV2.S. The humoral immune response was higher in healthy adults aged ≤65 years than in frail individuals above 65 years (**Table 1**), with the notable exception of Vaxzevria recipients. The HV of healthy adults induced antibody levels lower only than Spikevax vaccination.

**Table 1:** Demographic characteristics, baseline values and humoral immune response induced at T1 and T2 time-points by different vaccines in healthy adults and frail elderly individuals without any previously reported COVID-19 diagnosis.

	Comirnaty ≤65	Comirnaty >65	Spikevax ≤65	Spikevax >65	Vaxzevria≤ 65	Vaxzevria >65	Ad26.COV2.S≤65	Ad26.COV2.S>65	H V
***N=1401***	587	228	273	56	140	33	28	18	38
***Median age (IQR)***	46 (35 - 54.5)	71 (68 - 74)	43 (31 - 48)	71.5 (69 - 74.25)	61 (51 - 63)	68 (67 - 79)	63 (62 - 64.25)	68.5 (66.25 - 70)	51 (44.25 - 54.75)
***% Female***	55.37	46.49	59.71	39.29	57.14	51.52	67.86	50	42.11
***% T0 > 33.8***	3.58	3.95	5.86	7.14	5.71	0	14.29	5.56	2.63
***% T1 > 33.8***	99.83	96.93	100	89.29	98.57	100	89.29	77.78	100
***% T2 > 33.8***	99.83	93.42	100	83.93	91.43	96.97	67.86	66.67	100
***GMT T0 (Range)***	5.51 (4.81 - 315)	5.81 (4.81 - 1020)	6.24 (4.81 - 596)	7.05 (4.81 - 212)	5.96 (4.81 - 822)	5.04 (4.81 - 23)	7.65 (4.81 - 195)	5.73 (4.81 - 111)	5.34 (4.81 - 47.1)
***GMT T1 (Range)***	2120 (16.6 - 24900)	1040.1 (4.81 - 35300)	3777.1 (49.7 - 40100)	1084.47 (4.81 - 41000)	477.34 (17.1 - 3730)	658.66 (47.5 - 6780)	152.07 (19.2 - 13600)	77.56 (25.1 - 1030)	2566.02 (273 - 12800)
***GMT T2 (range)***	495.1 (33.2 - 20500)	268.66 (4.81 - 29400)	1084.92 (48.4 - 21100)	257.94 (4.81 - 22300)	189.17 (10.9 - 2310)	222.21 (29 - 5280)	115.8 (10.7 - 4370)	72.36 (7.77 - 3680)	820.87 (181 - 2580)

**Table 2:** Demographic characteristics, baseline values and humoral immune response induced at T1 and T2 time-points by different vaccines in healthy adults and frail elderly individuals with previously reported COVID-19 diagnosis.

	Ex-COVID1-dose <65	Ex-COVID2-dose <65	Ex-COVID1-dose ≥65	Ex-COVID2-dose ≥65
**N=118**	51	14	34	19
**Median age (IQR)**	51 (45 - 57)	68 (66 - 71.75)	47 (34 - 55)	76 (72.5 - 78.5)
**% Female**	56.86	50	47.06	42.11
**% T0 > 33.8**	94.12	92.86	73.53	94.74
**% T1 > 33.8**	100	100	100	100
**% T2 > 33.8**	100	92.86	100	100
**GMT T0 (Range)**	126.6 (4.81 - 2080)	246.61 (4.81 - 681)	53.43 (4.81 - 618)	102.22 (4.81 - 711)
**GMT T1 (Range)**	5847.75 (1440 - 37600)	4856.54 (34.2 - 21300)	7121.97 (1450 - 33800)	7627.06 (186 - 40200)
**GMT T2 (range)**	1556.36 (262 - 22800)	1667.61 (19.2 - 16100)	2622.08 (688 - 29400)	3804.02 (36.8 - 39800)

Among ex-COVID subjects, those who had two doses reached the highest GMT at T1 and T2. Humoral immune responses were consistently high in the frail elderly group (**Table 2**). Out of the 1,519 subjects analysed, 1,383 were anti-N IgG negative at baseline. Of those, 33 (2.4%) became positive at T1 or T2, an indicator of a possible infection.

According to the log-linear regression (**Table 3**), GMT at one month was expected to increase by 96.5% (95% CI: 73.0-123.2) in healthy adults compared to frail elderly. Taking as reference Spikevax, GMT at one month was expected to decrease by 82.8% (95% CI: 79.3-85.7, pvalue < 0.001), 95.8% (95% CI: 94.3-97.0, pvalue < 0.001), 36.5% (95% CI: 27.8-44.2, pvalue < 0.001), and 21.3% (95% CI: -10.6-43.8, pvalue=0.169) respectively among individuals who received Vaxzevria, Ad26.COV2.S, Comirnaty, and the heterologous vaccination, once the analysis is corrected for the other covariates. Finally, there was no significant difference in the GMT between male and female participants (p value 0.392).

**Table 3:** Average percentage variation of the GMT at T1 with respect to type of vaccine, age group, the response at T0 and sex and 95% confidence intervals, obtained as the exponential of the estimated coefficient of the log-linear regression model minus 1.

Covariates	Average percentage variation (95% CI)	p value
**Vaxzevria vs Spikevax**	-82.80% (-85.71; -79.31)	<0.001
**Ad26.COV2.S vs Spikevax**	-95.84% (-96.95; -94.32)	<0.001
**Heterologous vs Spikevax**	-21.32% (-43.77; 10.64)	0.169
**Comirnaty vs Spikevax**	-36.53% (-44.24; -27.76)	<0.001
**IgG anti-S T0 > 33.8 BAU/ml vs IgG anti-S T0 < 33.8BAU/mL**	294.27% (206.26; 407.58)	<0.001
**Healthy adults vs frail elderly**	98.82% (75.38; 125.39)	<0.001
**Male vs Female**	-4.53% (-14.15; 6.16)	0.392

As shown in **Table 4**, among healthy adults, the relative decrease in the GMT between 1 and 6 months was lower for subjects who received Vaxzevria (60.4%, p value relative to the test on differences with respect to Spikevax <0.001), the heterologous vaccination (68.0%, pvalue = 0.431), and Ad26.COV2.S (23.9%, pvalue <0.001), with respect to Spikevax (71.3%), while it was higher for subjects who received Comirnaty (76.7%, pvalue <0.001) with respect to Spikevax.

**Table 4:** Estimated fixed effect of the mixed effect log-linear model on the GMT with time, type of vaccine, a dichotomous variable indicating whether IgG anti-S T0 > 33.8 BAU/ml, and age group as covariates, and an interaction term between time and type of vaccine.

	Healthy adults			Frail elderly		
	Value	Std.Error	p-value	Value	Std.Error	p-value
**Intercept**	8,164	0,053	<0.001	6,819	0,191	<0.001
**Time T2 vs T1**	-1,247	0,048	<0.001	-1,436	0,156	<0.001
**Comirnaty vs Spikevax**	-0,549	0,064	<0.001	0,034	0,212	0,872
**Heterologous vs Spikevax**	-0,347	0,151	0,022	–	–	–
**Ad26.COV2.S vs Spikevax**	-3,317	0,173	<0.001	-2,600	0,385	<0.001
**Vaxzevria vs Spikevax**	-2,067	0,091	<0.001	-0,329	0,312	0,293
**IgG anti-S T0 > 33.8 BAU/ml vs IgG anti-S T0 < 33.8BAU/mL**	1,238	0,113	<0.001	2,375	0,355	<0.001
Interaction term: time T2 * Comirnaty	-0,207	0,058	<0.001	0,082	0,174	0,636
Interaction term: time T2 * Heterologous	0,108	0,137	0,431	–	–	–
Interaction term: time T2 * Ad26.COV2.S	0,975	0,157	<0.001	1,367	0,317	<0.001
Interaction term: time T2 * Vaxzevria	0,322	0,082	<0.001	0,350	0,256	0,174

*Since Spikevax is the reference, to obtain the relative decrease at T2 (vs T1) we exponentiate the time coefficient and substract 1. To obtain the relative decrease at T2 (vs T1) for the other vaccines, we first exponentiate the sum of the time coefficient and the relative interaction term and then substract 1.

Among frail elderly, the relative decrease in the GMT between 1 and 6 months was lower for subjects who received Ad26.COV2.S (6.7%) when compared with Spikevax (76.2%, pvalue relative to the test on differences < 0.001). On the other side, there was no significant difference in the variation of the GMT at consequent times between Comirnaty (74.2%) or Vaxzevria (66.3%) with respect to Spikevax (pvalues respectively 0.636 and 0.174).

### SARS-CoV-2 neutralization assays

**Figure 2:**
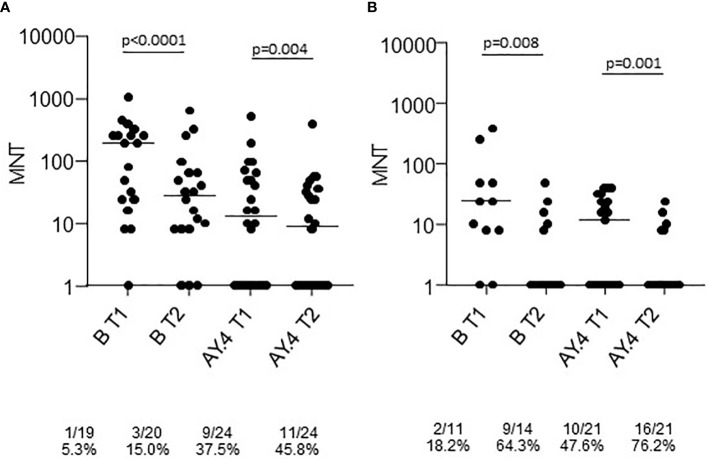
Microneutralization titers of T1 and T2 sera from COVID-19 naïve subjects vaccinated with Comirnaty. Sera from healthy adults **(A)** and frail elderly subjects **(B)** vaccinated with the Comirnaty vaccine were used to neutralize reference SARS-CoV-2 strain (B) and a Delta strain (AY.4). Individual MNT are reported together with median values. Non-neutralizing sera (MNT<8) are placed on the x-axis; frequencies of non-neutralizing sera are indicated below the graphs. Statistical differences among strains were calculated by the Kruskall-Wallis test; differences among time-points were calculated with the Wilcoxon test.

Sera collected at T1 and T2 from a representative sample of healthy adults ≤65 years and frail elderly individuals >65 years who received the Comirnaty vaccine and were COVID-19 naïve were randomly selected for virus neutralization assays against the B (reference), and the AY.4 (Delta) strains (**Figure 2**). Comparison of sera neutralizing activity showed a higher median MNT against B as compared to AY.4 [MNT median, interquartile range (IQR): healthy adults T1 B 129 (24;256) vs T1 AY.4 13 (<8; 60); healthy adults T2 B 28 (8;64) vs T2 AY.4 9 (<8; 35). The neutralization activity against both viral strains significantly declined at T2. When comparing frail elderly subjects with healthy adults, we found that the in the former group significantly higher percentages of individuals had lost neutralizing activity against the B and the AY.4 strains at T2 (64.3% vs 15.0% sera not neutralizing the B strain, elderly vs adults; 76.2% vs 45.8% sera not neutralizing the Delta AY.4 strain, elderly vs adults).

**Figure 3:**
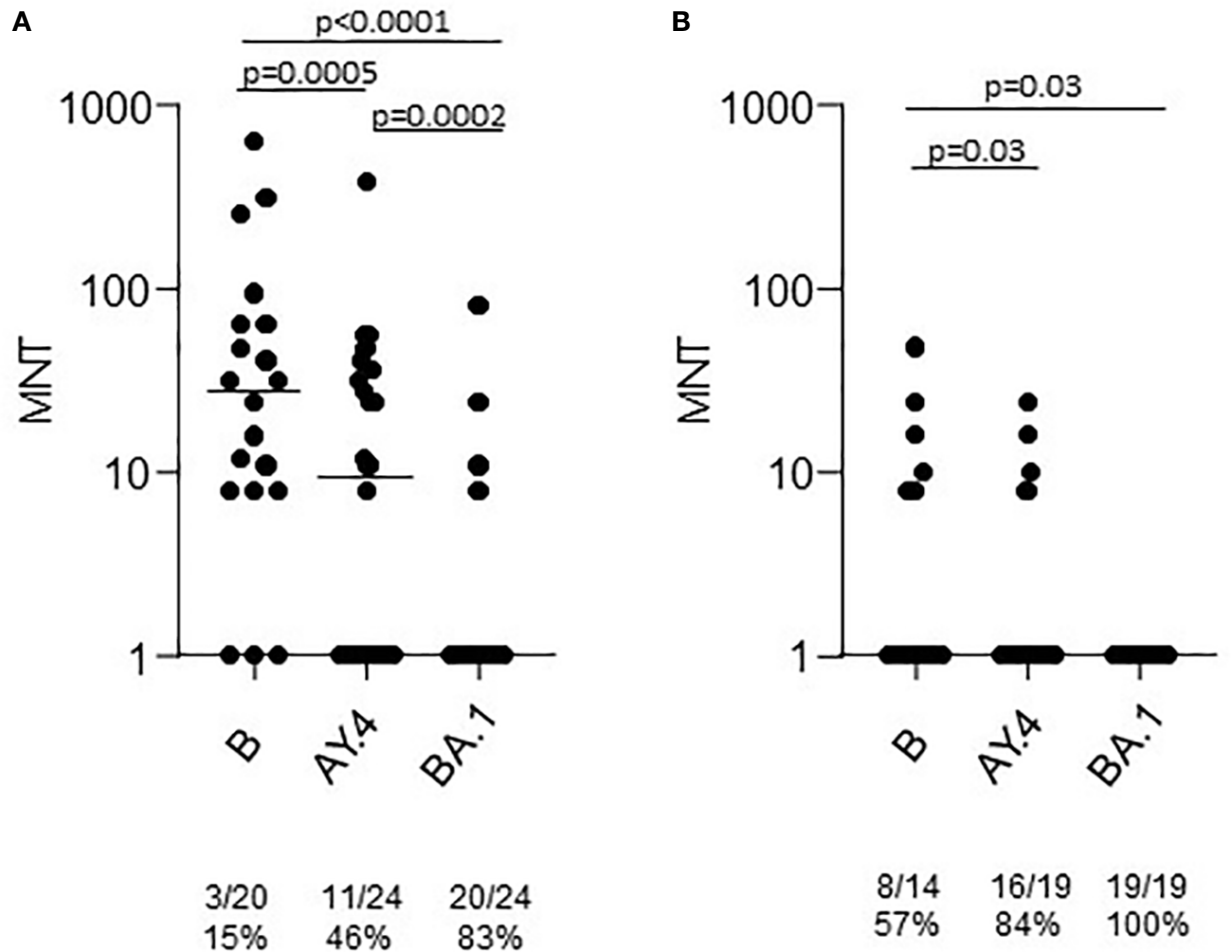
Microneutralization titers of T2 sera from COVID-19 naïve subjects vaccinated with Comirnaty. Sera from healthy adults **(A)** and frail elderly subjects **(B)** vaccinated with the Comirnaty vaccine were used to neutralize reference SARS-CoV-2 strain (B), a Delta strain (AY.4) and an Omicron strain (BA.1). Individual MNT are reported together with median values. Non-neutralizing sera (MNT<8) are placed on the x-axis; frequencies of non-neutralizing sera are indicated below the graphs. Statistical differences among strains were calculated by the Kruskall-Wallis test.

A group of the T2 sera was tested in the neutralization assays against the Omicron BA.1 strain. The results showed a statistically significant lower neutralizing activity (**Figure 3**). At T2 only 16% of sera from healthy adults had a neutralizing activity against BA.1. None of the sera from the > 65 group was able to neutralize the virus.

**Figure 4:**
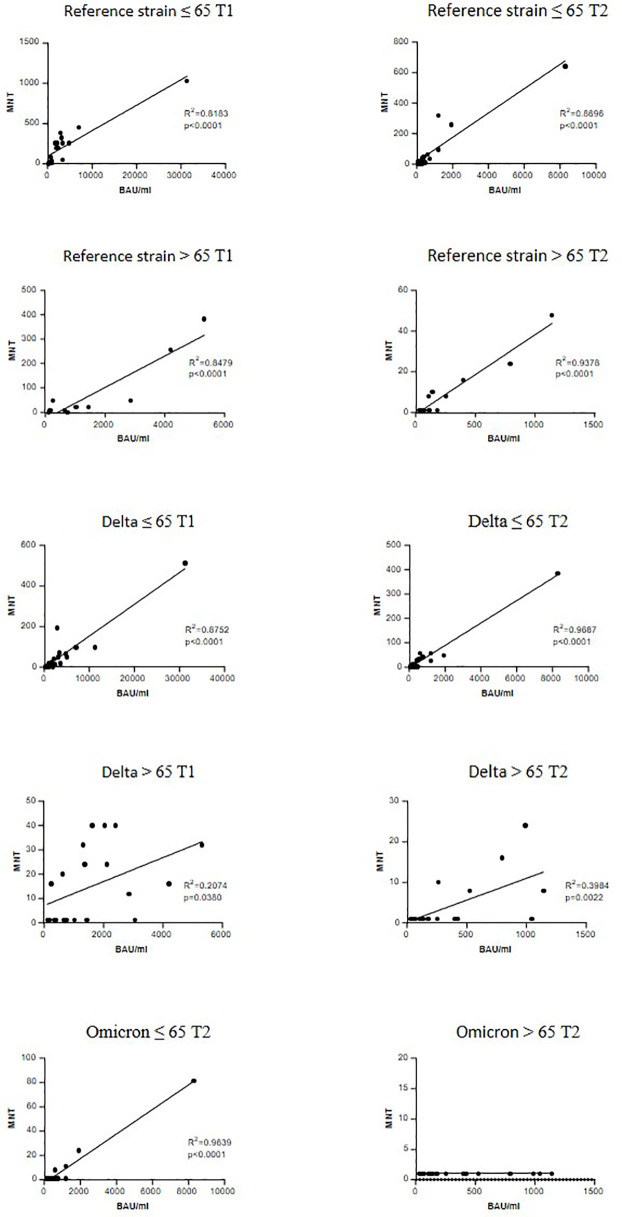
Correlation between anti-trimeric Spike IgG titers and serum neutralization activity. Linear regression correlating the levels of anti-trimeric S IgG with MNT values against reference SARS-CoV-2 strain (B), a Delta strain (AY.4) and an Omicron (BA.1). r and P values are shown.

A significant correlation between anti-trimeric S IgG levels and MNT against the reference strain was found in both age groups and at T1 and T2 (**Figure 4**). When considering the VOCs, serum IgG concentrations showed a good correlation with MNT among adults ≤65 years, but much lesser in the frail elderly group, suggesting that older individuals respond to vaccination with lower IgG titers and their antibodies have a reduced neutralizing activity.

### T-cell mediated immune response to vaccination

**Figure 5:**
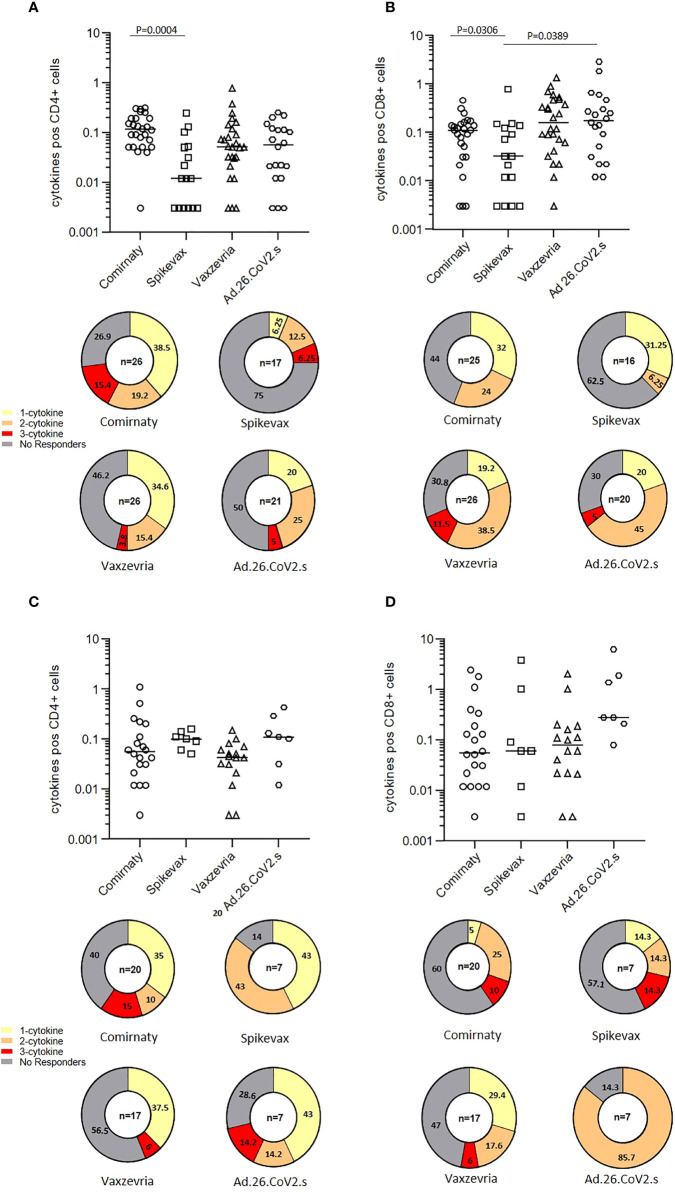
T-cell mediated immune response in COVID-19 vaccinees 6 months after first immunization. The frequencies of CD4+ and CD8+ T cells producing IFN-γ, TNFα and IL-12 in healthy adults **(A, B)** and frail elderly subjects **(C, D)** in response to *in vitro* stimulation with Spike are shown as total cytokine response. Statistical differences among types of vaccine were calculated by the Wilcoxon test. Pie diagrams show the frequencies of non-responding T cells or T cells producing 1 to 3 cytokines simultaneously.

The frequencies of CD4+ and CD8+ T cells producing IFN-γ, TNF-α and IL-2 in response to SARS-CoV-2 Spike peptides were measured in randomly selected COVID-19 naïve healthy adults six months after the first vaccine dose. Total cytokine response showed that among healthy adults, Spikevax recipients were those with the lower percentage of responders, i.e. individuals reacting to Spike antigenic stimulation with the production of at least one of the 3 cytokines analysed, both in CD4+ and CD8+ T cells (**Figures 5A, B**). Comirnaty vaccinees tended to induce preferentially CD4+ T cells responses whereas subjects vaccinated with adenovirus vectored vaccines had a preferential activation of CD8+ T cells. In COVID-19 naïve frail elderly subjects, Comirnaty and Vaxzevria vaccines induced a poorer response as compared to healthy adults, while Spikevax and Ad.26.COV2.S induced response was higher (**Figures 5C, D**). Individual cytokine responses are shown in **Supplementary Figure 4**. Most IgG low-responders, i.e. subjects with a IgG titer lower than 100 BAU/ml at T1, were able to mount a detectable T-cell response at T2 (**Supplementary Figure 4**, indicated by the grey-filled symbols). Overall, T-cell responses were not impaired in elderly subjects. The Ad.26.COV2.S vaccine, which ranked in last place as far as concerned the humoral response, induced a good T-cell response, especially in the >65 years group.

### B-cell memory response to vaccination

**Figure 6:**
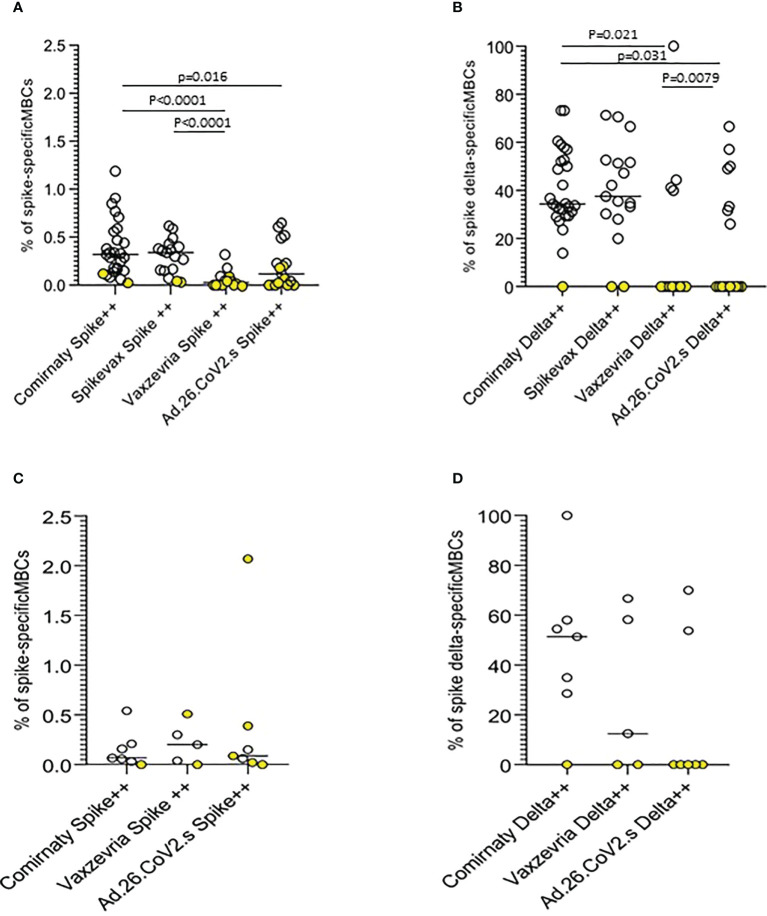
Anti-Spike specific B-cell memory responses in COVID-19 vaccinees 6 months after first immunization. The frequencies of memory B cells displaying high specificity towards the ancestral Spike antigen and the frequencies of ancestral Spike-specific memory B cells highly specific towards Delta (B.1.617.2) Spike antigen in healthy adults **(A, B)** and frail elderly subjects **(C, D)** are shown. Statistical differences were calculated by the Wilcoxon test.

Anti-Spike specific B-cell memory responses were assayed at the T2 time-point on a sample of 90 COVID-19 naïve vaccinated subjects, mostly healthy adults ≤65 years. Memory B cell (MBC) frequencies were higher in mRNA vaccine recipients. Ad.26.COV2.S vaccinees displayed higher frequencies than subjects vaccinated with the two-dose Vaxzevria vaccine (**Figures 6A, C**). mRNA vaccines induced MBCs with a broader repertoire, also able to recognize the Delta spike, on average approximately 40% of MBCs specific for ancestral SARS-CoV-2 Spike have specificity for the AY.4 Spike (**Figures 6B, D**). In frail elderly subjects vaccinated with Comirnaty, Vaxzevria and Ad.26.CoV2.S. Frequencies of Spike-specific MBC were lower as compared to healthy adults, although with a few outliers. Comirnaty vaccinees displayed a broader MBC repertoire with high affinity binding to Delta Spike.

## Discussion

Few studies compared immunogenicity of different COVID-19 vaccines and to date there has been limited head-to-head evaluation of immune responses to different types of COVID-19 vaccines (e.g. mRNA vs adenovirus-vectored vaccines) (21). The novelty of the present study relies on the comparison of different vaccine-induced immune components among healthy adults and frail elderly individuals.

The analysis of the humoral immune response showed a hierarchy in anti-S IgG inducing capacity either at one month and six months from the primary vaccine series, with Spikevax > Comirnaty >Vaxzevria > Ad.26.CoV2.S. Frail elderly subjects older than 65 and with at least two co-morbidities responded to the vaccination with lower amount of antibody than the younger counterpart. The decline of anti-trimeric Spike IgG between T1 and T2 was reduced in those vaccinated with Vaxzevria and significantly reduced in those vaccinated with Ad.26.CoV2.S, independently of their age, as found in the mixed effect model (**Table 4**). In this respect, data on antibody decline in Ad.26.CoV2.S vaccinated subjects were in line with results published by Zhang and colleagues (21). At six months from the vaccination, still most of the enrolled individuals had antibody levels above the positivity threshold of the assay used. These overall findings are consistent with previous reports on COVID-19 vaccines (22, 23). The performance of the heterologous vaccination approach was remarkable confirming previous study showing the benefits of this approach (24–26).

A small fraction of enrolled individuals had a COVID-19 diagnosis prior of vaccination. A previous SARS-CoV-2 infection was associated with higher antibody levels, suggesting that prior infection history may increase protection from vaccination (27). The potentiating effect of a previous infection is not surprising and might be attributed to the so-called hybrid immunity (28). We found that one single vaccine dose in ex-COVID subjects induces similar antibody levels as compared to a two-dose schedule. Remarkably, although uncertainty around the antibody response at different times is large due to small sample sizes, frail elderly ex-COVID subjects were not lesser able to mount a humoral response to the vaccination than healthy adults. This finding may be biased by a confounding factor related to the severity of symptoms after infection, but unfortunately information on the severity of symptoms after the infection is not available. Nonetheless, according to a recent study among nursing home residents, a marked increase of humoral immune response to mRNA COVID-19 vaccines was found in those with a previous history of SARS-CoV-2 infection (29). Even though serum levels of IgG induced by vaccination represents a key marker of vaccine performance, the functional quality of vaccine-induced antibody needs to be assessed and a clear correlate of protection is not available so far, although it has been suggested that neutralizing antibodies induced by COVID-19 vaccination correlate with protection (10, 14). When we measured neutralizing antibodies against the reference SARS-CoV-2 strain (lineage B) in Comirnaty vaccinees, we found that they were detectable in 95% of healthy adults and in 85% of frail elderly subjects one month after the second vaccine dose (T1). Similarly to anti-trimeric IgG, neutralizing antibodies were significantly higher in the ≤ 65 years group and declined six months after vaccination. Sera tested against the AY.4 (Delta) strain had significantly lower titers as compared to the B strain in both age groups. From the end of 2021, the highly mutated BA.1 strain (Omicron) started to circulate in Italy (30). We found that six months after the first dose of Comirnaty, only few sera from healthy adults and none from frail elderly subjects had the capability to neutralize this variant, as already observed in other settings (31, 32).

An interesting result was found when we correlated the neutralizing activity with anti-trimeric Spike IgG levels. The correlation with neutralizing activity against SARS-CoV-2 B and AY.4 strains was significant in both younger and older adults at T1 and T2. There was no correlation between anti-trimeric Spike IgG and antibodies with neutralizing activity against BA.1 in the > 65 group at T2. This could be possibly related to immune ageing with consequent reduced size and function of the germinal centre response (33).

T-cell mediated immune responses were analysed at the T2 time-point in subjects who received each of the 4 vaccines under comparison. Data on T-cell responses were not correlated to vaccine-induced antibody response, indeed, the Spikevax vaccine was poor while the Ad.26.COV2.S was efficient in inducing T-cell immunity at 6 months from vaccination. Moreover, humoral low-responders, that is those with IgG levels below 100 BAU/ml at T1, were generally able to mount a good T-cell response. Compared to already published data, our results on T-cell responses confirm the development of a persistent T-cell memory response in Comirnaty, Vaxzevria and Ad.26.COV2.S vaccinated individuals (21, 34–38). At difference from those study, we found poorer T-cell responses induced by Spikevax as compared to other vaccines, however it should be pointed out that the in study by Zhang et al. and by Goel et al. the activation induced marker (AIM) assay was used to assess T-cell response in vaccinated subjects.

The last aspect of vaccine-induced immunity analysed in the present study concerns memory B cells. In contrast to serum antibodies, memory B cell responses after COVID-19 vaccination are long-lived and play an important role in protection by rapidly reacting to infection with the production of IgG antibodies in the serum and at the site of viral entry (16, 17). High affinity B-cell memory are induced at the T2 time-point in healthy adults by mRNA vaccines at higher frequencies as compared to adenovirus vectored vaccines. Thus, mRNA vaccines are stronger inducer of B cell responses, measured by specific antibody levels and frequency of memory B cells, than Vaxzevria and Ad26.COV2.S six months after the first vaccine dose. Most MBC from Comirnaty and Spikevax vaccinees were able to recognize Delta Spike. Vaccinees not able to recognize Delta Spike generally had low frequencies of MBC with high affinity towards WT Spike suggesting that the germinal center reaction is impaired in these subjects (39).

Our study has some limitations. The numbers of enrolled individuals in the Vaxzevria > 65 group and in both Ad26.COV2.S groups are smaller; the study protocol did not include subject under 18 years of age and especially children for which humoral and cellular responses are not available for evaluation. Moreover, it is important to remark that data obtained so far, and here reported, include the response to primary two-doses vaccination and are related to the first part of an immunological monitoring study which is currently still ongoing. In this regard, it has been shown that a third dose strongly boosts the antibody responses in older individuals also against some circulating VOCs (12, 13).

Overall, the data demonstrated that the COVID-19 vaccines in elderly subjects are immunogenic, despite immune-ageing and frailty. As already described (11), we found that older individuals have lower neutralizing titres against SARS-CoV-2 than younger adults, however most of them were able to mount a T-cell immune response. Worth of note, analysis of humoral immune responses shows greater differences among vaccines than T-cell immune responses. Heterologous vaccination and heterologous combination of infection plus vaccination strongly improves antibody response, also against some of the known VOCs.

High affinity B-cell memory are induced at the T2 time-point in healthy adults by mRNA vaccines at higher frequencies as compared to adenovirus vectored vaccines. Thus, mRNA vaccines are stronger inducer of B cell responses as drivers of immunological memory response.

## Data availability statement

The raw data supporting the conclusions of this article will be made available by the authors, without undue reservation.

## Ethics statement

The study was approved by the Ethical Committee of the National Institute for Infectious Diseases Lazzaro Spallanzani (Parere n. 271 del Registro delle Sperimentazioni, 04.02.2021 and subsequent amendments). Thereafter, approval was given by local ethic committees (Comitato Etico dell’Università La Sapienza, Rif n.6358; Ligurian regional ethics committee, n. CER Liguria 164/2021; Ethics Committee for Clinical Trials of the Province of Padua n. 5101/U6n/21, 17.06.2021; CE AVEC: 399/2021/Sper/AOUBo; Comitato Etico Milano Area 2 c N. 1473, 13/05/2021; Comitato Etico Palermo 1, 18/02/2021; Comitato Etico Area 1 Puglia, 10/02/2021). The patients/participants provided their written informed consent to participate in this study.

## Author contributions

PS: conceived the study. GF, FT, and PS: designed the study, analysed the data, and wrote the manuscript. GF, PL, and IS: performed anti-S and anti-N IgG immunoassays and neutralizing antibody assays. SF and AM: performed SARS-CoV-2 neutralizing antibody assays. TB, AB, FB, MP, FF, RG, VL, AO, and VR: managed biological samples collection and handling and performed anti-N IgG immunoassays. IS, EO, GF, and RG: performed T-cell response experiments. RC, EPM: performed memory B-cell response experiments. SA, GA, VB, PC, AG, GI, TL, CM, RP, and VR: contributed to study design provided samples and data and revised the manuscript. AP, SM, and SB critically revised the manuscript. All the authors contributed to editing of the manuscript.

## Conflict of interest

The authors declare that in this study the research was conducted in the absence of any commercial or financial relationships that could be construed as a potential conflict of interest.

## Publisher’s note

All claims expressed in this article are solely those of the authors and do not necessarily represent those of their affiliated organizations, or those of the publisher, the editors and the reviewers. Any product that may be evaluated in this article, or claim that may be made by its manufacturer, is not guaranteed or endorsed by the publisher.
