# Supplementary material for: Evaluation of humoral and cellular response to four vaccines against COVID-19 in different age groups: A longitudinal study
Source: Front Immunol. 2022 Oct 31;13:1021396. doi: 10.3389/fimmu.2022.1021396 (PMC9661524; doi:10.3389/fimmu.2022.1021396)
Supplement: Supplementary file 1 [file Table_1.docx]

**Supplememtary Table 1.** List of comorbidities associated with increased risk of severe COVID-19

| Ischemic Heart Disease |
| --- |
| Atrial Fibrillation |
| Heart Failure |
| Stroke |
| Arterial Hypertension |
| Diabetes |
| Dementia |
| COPD |
| Active Cancer (last 5 years) |
| Chronic Epathopathy |
| Chronic Renal Failure |
| Autoimmune Disease |
| Obesity |

| Institution | City and Region |
| --- | --- |
| Policlinico Umberto 1, Sapienza University | Rome, Lazio |
| Ospedale Maggiore Policlinico, University of Milano, Istituto Nazionale di Genetica Molecolare | Milan, Lombardy |
| Policlinico Sant’Orsola, University of Bologna | Bologna, Emilia-Romagna |
| ASST Ovest Milanese | Legnano, Lombardy |
| Policlinico Riuniti, University of Foggia | Foggia, Apulia |
| Policlinico San Martino, University of Genoa | Genova, Liguria |
| Policlinico Paolo Giaccone, University of Palermo | Palermo, Sicily |
| University of Padova | Padua, Veneto |

**Supplementary Table 2.** List of collaborating centres involved in the study
