# Supplementary figures and images for: Evaluation of humoral and cellular response to four vaccines against COVID-19 in different age groups: A longitudinal study
Source: Front Immunol. 2022 Oct 31;13:1021396. doi: 10.3389/fimmu.2022.1021396 (PMC9661524; doi:10.3389/fimmu.2022.1021396)

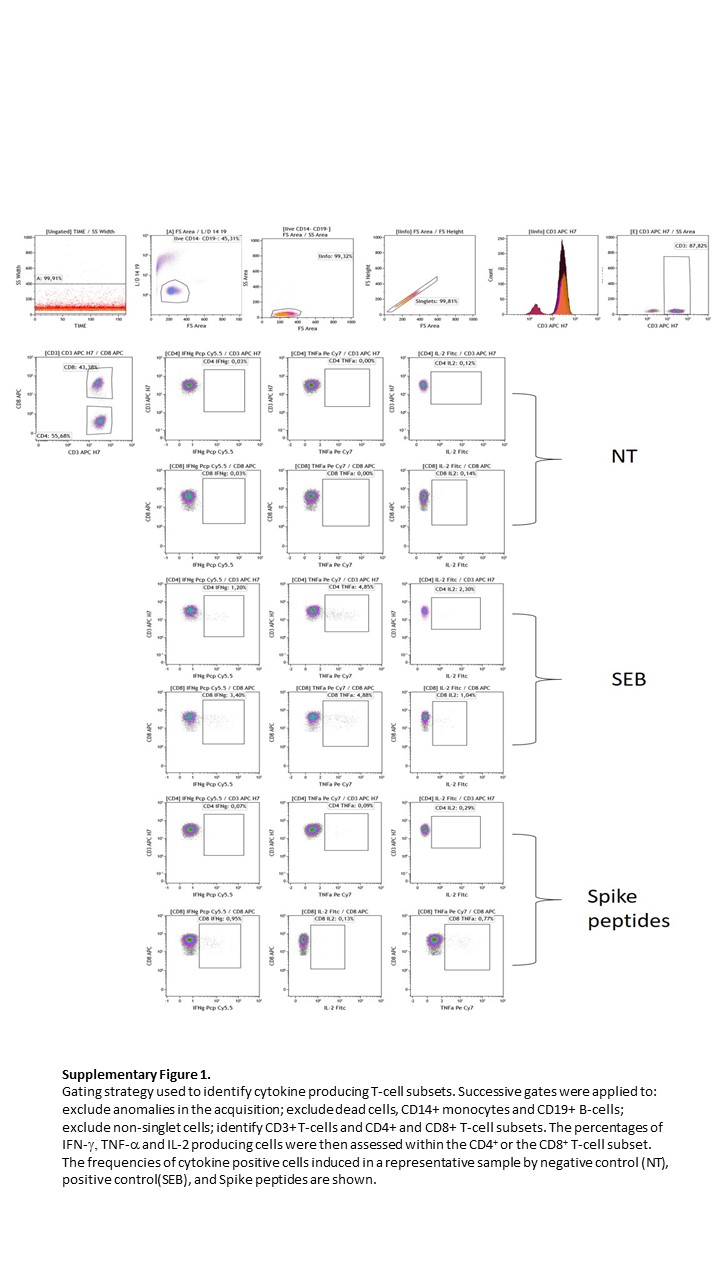

Supplement: Supplementary file 2 [file Image_1.jpeg]

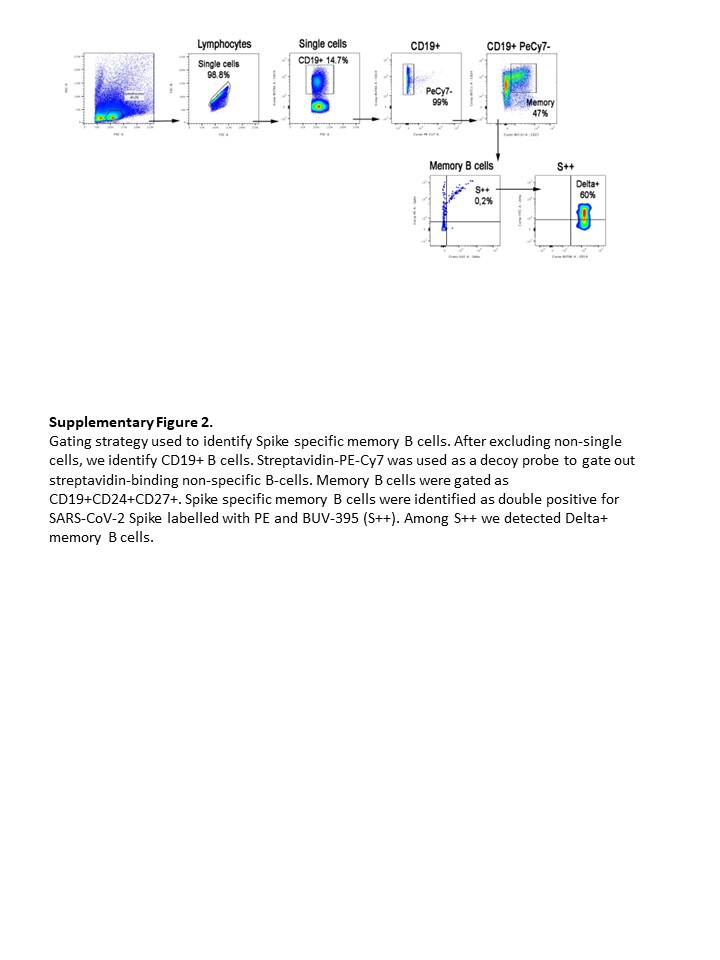

Supplement: Supplementary file 3 [file Image_2.jpeg]

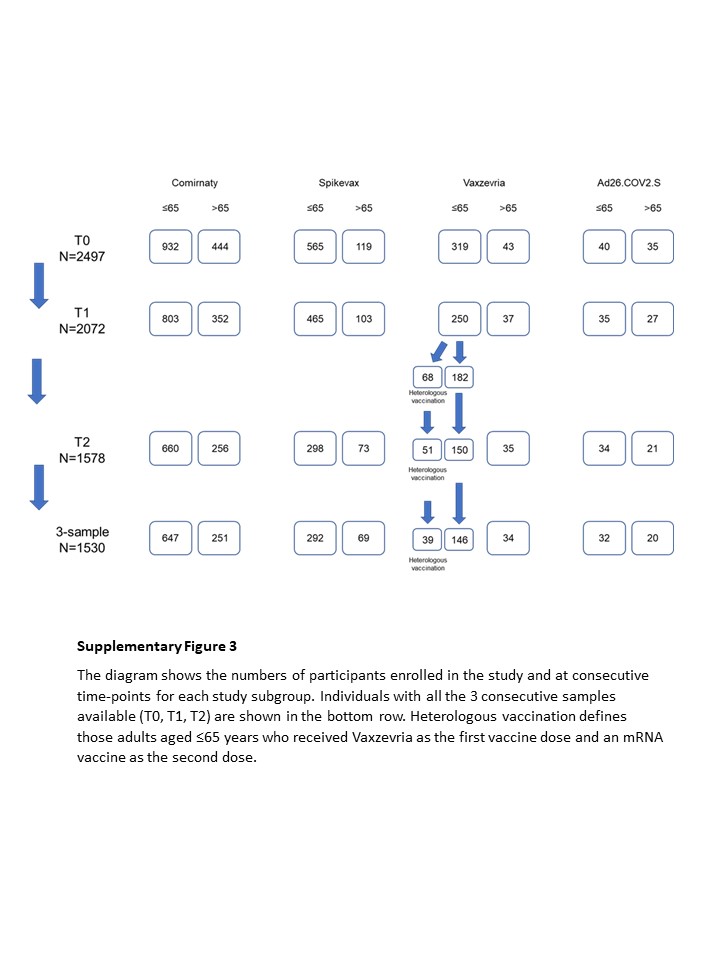

Supplement: Supplementary file 4 [file Image_3.jpeg]

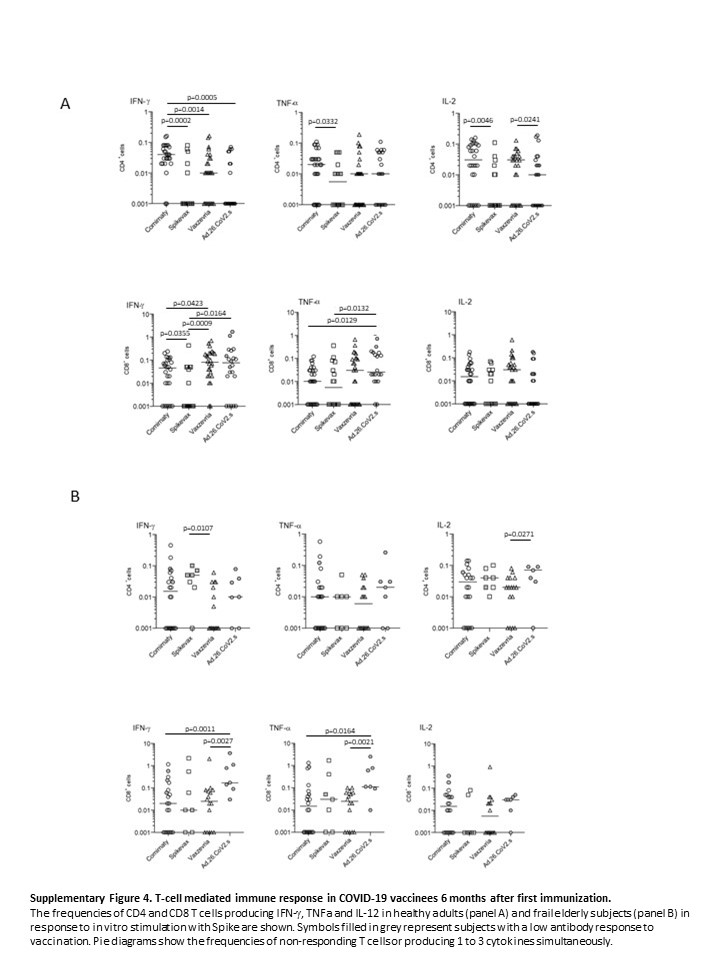

Supplement: Supplementary file 5 [file Image_4.jpeg]
